# Cholinergic Regulation of Hippocampal Theta Rhythm

**DOI:** 10.3390/biomedicines10040745

**Published:** 2022-03-23

**Authors:** Zhenglin Gu, Jerrel L. Yakel

**Affiliations:** Neurobiology Laboratory, National Institute of Environmental Health Sciences, National Institutes of Health, Department of Health and Human Services, Research Triangle Park, NC 27709, USA

**Keywords:** acetylcholine, hippocampus, theta rhythm

## Abstract

Cholinergic regulation of hippocampal theta rhythm has been proposed as one of the central mechanisms underlying hippocampal functions including spatial memory encoding. However, cholinergic transmission has been traditionally associated with atropine-sensitive type II hippocampal theta oscillations that occur during alert immobility or in urethane-anesthetized animals. The role of cholinergic regulation of type I theta oscillations in behaving animals is much less clear. Recent studies strongly suggest that both cholinergic muscarinic and nicotinic receptors do actively regulate type I hippocampal theta oscillations and thus provide the cholinergic mechanism for theta-associated hippocampal learning. Septal cholinergic activation can regulate hippocampal circuit and theta expression either through direct septohippocampal cholinergic projections, or through septal glutamatergic and GABAergic neurons, that can precisely entrain hippocampal theta rhythmicity.

## 1. Introduction

The hippocampus has been widely accepted as the brain region for memory encoding and short-term memory storage. The hippocampus receives major excitatory inputs from entorhinal cortex and sends the major output back to the entorhinal cortex [1,2]. The hippocampus also receives extensive cholinergic and GABAergic inputs from medial septum and diagonal band of Broca (MSDB) [2]. Muscarinic acetylcholine receptor antagonist scopolamine has been long known to impair memory encoding. Accordingly, cholinergic regulation of hippocampal activity has been proposed as a crucial mechanism for memory encoding [3,4]. One featured activity pattern in the hippocampus is theta oscillations. Theta oscillations are large rhythmic fluctuations of the field potential in the hippocampus and many hippocampus-associated brain regions, mostly during active exploration. Due to the observation of phase precession of individual place cell firing relative to the theta phase when the animal is approaching and passing through a place field, theta oscillations have been proposed as a vehicle for encoding the sequence of place cells in spatial memory and potentially the sequence of events in episodic memory [5,6,7,8,9]. Cholinergic transmission is also closely related to theta oscillations [10,11]. Therefore, cholinergic regulation of theta oscillations is of great importance in hippocampal functions especially in memory encoding. However, cholinergic transmission is traditionally more closely linked to the type II theta under urethane anesthesia and alert immobility, which is also called atropine-sensitive theta, since type II theta is eliminated by the muscarinic receptor antagonist atropine (Table 1). On the other hand, the type I theta oscillation that occurs during active exploration, which is supposed to be the one involved in memory encoding, is largely atropine resistant (Table 1) [11,12]. This makes it difficult to explain the potential cholinergic role in memory encoding through regulation of hippocampal theta oscillation in behaving animals. Recent studies suggest that even though atropine does not eliminate type I theta as it does to type II theta, cholinergic transmission indeed can still actively regulate certain aspects of type I theta oscillations and subsequent behavioral outcomes through both muscarinic and nicotinic receptors [13,14].

Septal cholinergic and GABAergic inputs to the hippocampus have been traditionally deemed as the pacemakers of theta oscillations, providing rhythmic excitatory and inhibitory hippocampal inputs, respectively [12]. However, recent optogenetic studies suggest that septal cholinergic activity had little direct effect on hippocampal theta rhythm [15,16]. Instead, septal parvalbumin-positive interneurons can directly pace hippocampal theta rhythm [15,17]. Still, it is unlikely that individual septal interneurons pace hippocampal theta. Instead, it is more likely that the interneurons as a population play the pacemaker role as the timing of individual septal neuronal firing is too variable to consistently lead each theta cycle [18]. Even though cholinergic inputs do not directly pace theta rhythm, they can still regulate the intensity and/or the frequency of theta oscillations directly through septohippocampal cholinergic pathway or indirectly through septal local GABAergic and glutamatergic neurons that can precisely pace theta rhythm.

## 2. MSDB Cholinergic Neuronal Activities Correlate with Theta States

There is strong evidence supporting cholinergic involvement in not only type II theta but also type I theta. Several studies have observed elevated septal cholinergic firing rate or hippocampal acetylcholine (ACh) release during both type I and type II theta dominant behavioral states [3,19,20,21,22,23]. Microdialysis measurements of hippocampal acetylcholine levels in freely moving cats show a significant increase of ACh level during active waking and REM sleep over slow wave sleep baseline or quiet waking [20]. Additional microdialysis studies also found elevated hippocampal ACh levels in freely moving rats during active exploration [22,23]. A recent amperometry study that simultaneously monitored ACh level and local field potential in the dorsal hippocampus also uncovered a clear association between phasic ACh release and induced or spontaneous theta oscillations in urethane-anesthetized rats [19]. A more recent study using optical detection of an acetylcholine sensor fluorescent signal also shows a clear correlation between hippocampal ACh level and theta power in behaving mice [3]. Direct recordings from septal neurons also shows that medial septal cholinergic neuronal activities highly correlate with theta occurrence in freely moving mice [21]. Septal cholinergic neurons are highly active during theta dominant periods, such as active exploration and rapid eye movement (REM) sleep, while they are much less active during non-theta periods such as slow-wave sleep (SWS). However, optogenetic activation of septal cholinergic neurons had little effect on theta oscillations during either non-theta period or theta dominant periods, suggesting that cholinergic activation played a permissive role in theta generation and expression rather than as a driving force. Optogenetic activation of septal cholinergic neurons inhibited sharp wave ripples during slow-wave sleep, which is largely consistent with other studies [15,16]. These studies show that optogenetic activation of septal cholinergic neurons completely blocked sharp wave ripples and robustly enhanced theta oscillations in urethane-anesthetized mice but had less direct effect on type I theta in behaving mice. Yet cholinergic activation suppressed peri-theta events in both anesthetized and behaving mice and thus allowed theta to dominate [16]. Septal cholinergic activation can directly regulate hippocampal theta through elevated hippocampal ACh release and indirectly through the local septal circuit.

## 3. Cholinergic Regulation of Theta through Direct Septohippocampal Cholinergic Pathway

Both hippocampal muscarinic and nicotinic receptors may contribute to theta regulation [14,15,24,25,26,27,28]. Systemic administration of an α7 nicotinic ACh receptor (nAChR)-selective agonist significantly enhanced brainstem stimulation-induced hippocampal theta power in anaesthetized rats and mice [25,26]. Local hippocampal infusion of either muscarinic or α7 nAChR antagonists reduced peak theta power in freely moving mice, while ipsilateral entorhinal cortical infusion of a cocktail of cholinergic receptor antagonists had little effect on theta power, suggesting that the hippocampus but not entorhinal cortex was the primary target of cholinergic transmission in regulating theta oscillations [14,24]. Furthermore, cholinergic receptor subtype knockout studies suggest that mAChRs expressed in glutamatergic neurons (but not interneurons), and α7 nAChRs expressed in interneurons especially oriens lacunosum moleculare (OLM) interneurons (but not glutamatergic neurons), regulated theta oscillations [14,24]. OLM neurons are a subset of somatostatin-positive interneurons in the CA1 stratum oriens hippocampal layer that primarily target the distal dendrites of pyramidal neurons in stratum lacunosum–moleculare (SLM), overlapping with entorhinal cortical excitatory inputs. OLM neurons may play an important role in theta generation and learning and memory processes [29,30,31,32]. OLM neurons usually have larger α7 nAChR currents than pyramidal neurons and other hippocampal interneurons [14]. α7 nAChR activation on OLM interneurons can directly inhibit EC inputs in SLM but can enhance SC inputs through disinhibition [33]. In addition, α7 nAChR activation on OLM interneurons likely contribute to theta regulation through the disinhibition pathway [14]. Interestingly, direct optogenetic activation of ventral hippocampal OLM neurons can induce type II theta that can be blocked by systemic administration of atropine [34], providing a potential role for OLM neurons to coordinate mAChR and nAChR pathways in regulating theta oscillations. In vitro brain slice studies suggest that mAChR activation may primarily contribute to transient increases of theta power while α7 nAChR activation, together with mAChR activation, may promote synaptic plasticity and prime the network for theta generation by similar stimuli in the future [14]. Therefore, hippocampal cholinergic transmission may recruit different neuronal subpopulations through different receptor subtypes to regulate different aspects of theta oscillations.

Calcium imaging studies showed that calcium activities in dorsal CA1 pyramidal neurons are high during theta states including active exploration and REM sleep, and low during non-theta states including quiet wakefulness and slow wave sleep. Systemic or local hippocampal administrated mAChR antagonist scopolamine significantly reduced calcium activities in pyramidal neurons [35]. Higher calcium activities associated with theta states may promote synaptic plasticity and memory encoding [4], or place field stabilization [36]. Theta oscillations have been proposed as a mechanism for temporal coding due to phase precession and theta sequences of place cell firing [37,38]. Phase precession refers to the progressively earlier spiking time of a place cell relative to the theta phase when the animal traverses a place field. Accordingly, there can be several place cells firing sequentially in one theta cycle, representing the temporal order of the place fields the animal travels through. Systemic administration of mAChR antagonist scopolamine significantly impairs place cell phase precession [39,40]. Scopolamine significantly reduces the firing frequency of place cells to the same level as local field theta frequency, and thus eliminates the progressive phase precession. As such, the theta phase of individual place cell firing can no longer predict the position the animal travels [40]. Phase precession also depends on intact medial entorhinal cortical inputs to the hippocampus [41]. Scopolamine likely reduces phase precession through disrupting the entorhinal hippocampal interaction. However, theta sequences and place cell assemblies remained intact after the disruption of phase precession by scopolamine [39], suggesting differential mechanisms may underlie phase precession and theta sequence generation. Phase precession also occurred during the first lap on a novel linear track, but theta sequences were absent on the first lap and developed immediately afterwards and were stable once established [42]. Some studies show that place cell sequences formed in a novel spatial experience significantly correlates with spiking events before the novel experience, suggesting the place cell sequences formed during a novel experience result from the interplay of internal drives that likely arise from past experiences and external drives that come from the current novel experience [43,44]. Place cell sequences are more dynamic in the earlier stage and stabilize in the later stage. Taken together, cholinergic transmission may thus promote phase precession and the integration of constantly updated entorhinal cortical inputs during the whole course of an experience, but likely facilitates the formation and stabilization of theta sequences during the early stage of the experience. Once the theta sequences are established, they are no longer sensitive to cholinergic modulation. This is consistent with the general observation that cholinergic transmission is primarily involved in memory encoding but not memory retrieval [45]. It is also consistent with a brain slice study where cholinergic activation promotes synaptic plasticity and theta induction, but once theta was induced and stabilized it was no longer cholinergic sensitive [24].

## 4. Cholinergic Regulation of Theta through Septal GABAergic and Glutamatergic Neurons

MSDB is indispensable for both type I and type II theta generation and has been proposed as the host of theta pacemakers [46,47,48]. Three types of neurons, including fast-firing and burst-firing GABAergic, slow-firing cholinergic, and cluster-firing glutamatergic neurons, have been identified in the MSDB [49,50]. All have been shown to play crucial roles in theta generation in both type II and type I theta. Septal GABAergic and cholinergic have been traditionally thought to be the pacemakers of hippocampal theta oscillations [12], but recent optogenetic studies have clearly shown that while septal GABAergic neurons, especially PV neurons, can directly pace hippocampal theta rhythm [15,17], septal cholinergic activation only has a slight effect on hippocampal theta frequency in urethane-anesthetized mice [15]. Optogenetic activation of septal PV neurons with a stimulation range of 3–40 Hz induced hippocampal rhythmicity at exactly the stimulation frequency with the maximum theta power at about 10 Hz. Optogenetic activation of septal cholinergic neurons with the same stimulation range only resulted in a theta frequency range of 3.8 to 4.6 Hz [15]. Intraseptal infusion of carbachol elicited a higher frequency theta (6.8 Hz) in awake rats during non-theta states (such as alert immobility, chewing, lapping, and grooming) [51]. Intraseptal application of a muscarinic antagonist abolished cholinergic activation-induced theta, while intrahippocampal application of a combination of muscarinic and nicotinic antagonists also significantly reduced theta power [15], suggesting a crucial role of the septal local circuit in theta rhythm generation while hippocampal cholinergic activation more likely regulated theta expression. Intraseptal infusion of atropine also abolished vagal nerve stimulation-induced type II theta in rats [52]. Septal infusion of atropine not only abolished theta recorded during immobility, but also severely impaired the initiating movements in rats in defense of their food, suggesting atropine-sensitive theta is involved in the initiation of movements in response to sensory stimuli [53]. Intraseptal infusion of atropine also abolished theta in freely behaving cats by reducing the theta power and had little effect on frequency [54]. Both intraseptal and intrahippocampal administered atropine significantly reduced theta power in urethane-anesthetized rats [55]. In another study, hippocampal ACh level correlated with type II theta in urethane-anesthetized rats, but lagged behind theta initiation, suggesting that cholinergic regulation of hippocampal circuit is not critical in theta initiation, but rather works with theta to promote synaptic plasticity associated with learning and memory [19]. These studies strongly implicate the septal local circuit as the likely cholinergic target in type II theta initiation. In vitro slice experiments show that septal cholinergic activation depolarizes both PV neurons and other septal neurons but does not reliably induce action potentials [15], consistent with the observations that cholinergic activation does not directly pace theta frequency. Direct septo-hippocampal cholinergic input increased hippocampal inhibitory interneuron firing but reduced hippocampal pyramidal cell firing. By doing so, this pathway may increase the coupling of hippocampal pyramidal neuron firing time to the theta phase [15]. Septal cholinergic and GABAergic activation can work together to start the theta rhythm. GABAergic inputs to the hippocampus act to inhibit hippocampal theta-off cells (cells become virtually silent during theta), and cholinergic inputs provide the excitatory drive for hippocampal theta-on cells (cells with higher firing rates during theta) [56]. These events occur about 500 ms before the start of theta [47]. However, the theta oscillations likely occur in an interactive septo-hippocampal loop instead of being simply controlled by septal GABAergic neurons. It has been shown that intrahippocampally infused carbachol-induced type II theta in urethane-anesthetized rats can be abolished by septal inactivation [56]. Moreover, carbachol-induced rhythmic theta-like hippocampal oscillations were synchronized with rhythmic IPSPs that evoked rebound spiking in GABAergic, but not cholinergic and glutamatergic septal neurons. Therefore, rhythmic hippocampal activity can preferentially phase the spiking of septal GABAergic neurons [57]. These results suggest that hippocampal activity can also influence the rhythmicity of septal GABAergic neurons.

Other than septal GABAergic neurons, septal glutamatergic neurons can also regulate theta frequency. Rhythmic optogenetic activation of local septal glutamatergic neurons lineally entrained hippocampal type I theta frequency in behaving mice in the 6–10 Hz range but had little effect on peak theta power [58]. Septal glutamatergic neurons also projected to the hippocampus, but optogenetic activation of the glutamatergic hippocampal projection via the fornix did not significantly change theta frequency or power [58]. Moreover, intraseptal microinjection of an AMPA receptor antagonist reduced type I theta rhythm frequency with little effect on type I theta power [59], further supporting a role of septal glutamatergic activity in type I theta frequency regulation. Septal glutamatergic neurons can excite both local GABAergic and cholinergic neurons [60], but may preferentially target GABAergic neurons since direct optogenetic activation of septal glutamatergic neurons elicited postsynaptic EPSPs in both local GABAergic and cholinergic neurons, but only induced action potentials in a subset of GABAergic neurons and none of the cholinergic neurons [58]. Since cholinergic activation had little effect on directly pacing hippocampal theta frequency, the effect of glutamatergic pacing on theta frequency is likely through septal GABAergic neurons. On the other hand, septal glutamatergic neurons may have mediated the cholinergic effect at least in type II theta generation. Intraseptal microinjection of a glutamate AMPA receptor antagonist strongly inhibited type II theta that was indued by several means, including intraseptal infusion of carbachol [59] or physostigmine [61]. Even though the direct glutamatergic septohippocampal pathway has little effect on theta power and frequency, septal glutamatergic activities are closely related with locomotion. The firing of septal glutamatergic neurons not only initiates the transition from resting to locomotion, the firing rate and number of active neurons directly related to the speed and duration of locomotion by upregulating CA1 pyramidal excitability through oriens interneuron-mediated disinhibition [62]. Combined with their ability to modulate theta frequency through septal local circuit, septal glutamatergic neurons provide a potential mechanism to couple neuronal firing rates and theta oscillations to movement velocity [62]. It is currently not clear if septal cholinergic neurons also regulate this function.

## 5. Cholinergic Regulation of Theta Frequency

Although direct optogenetic activation of septal cholinergic neurons only had mild effect on type I theta (likely due to the already elevated cholinergic activation during theta states), septal cholinergic activation can still regulate theta through septal GABAergic and glutamatergic neurons, or through direct septohippocampal cholinergic pathway. Systemic or intraseptal administration of atropine mildly decreased type I theta frequency in freely moving rats [11,51]. The fine regulation of theta frequency by cholinergic transmission may have significant behavioral consequences. Theta frequency may be finely tuned to accommodate a variety of behavior performance. For example, theta frequency changes with speed, acceleration, and novel environment [63,64,65,66,67]. Indeed, systemically administered scopolamine significantly reduced speed modulation of theta frequency [68]. Septal infusion of carbachol induced continuous type II theta in awake rats and the theta frequency can further be upregulated by voluntary movement [51], suggesting the emergence of a form of theta that integrates both type I and type II theta components. Due to the differential theta frequency in type I and type II theta, cholinergic transmission could regulate theta frequency through regulating type II component in freely moving animals. If this is the case, then higher theta frequency would be expected after cholinergic blockade. Contradicting results have been reported. In one study, intraperitoneal injections of scopolamine shifted the hippocampal theta activity to a higher peak frequency in freely moving rats [69], while other studies showed that atropine decreased the frequency of type I theta in freely moving rats [11,51]. During novel environment exposure, theta frequency reduction [63] correlates with increased hippocampal ACh level [22,70]. This would be consistent with the notion that cholinergic activation could reduce theta frequency by upregulating type II component. However, direct evidence showing cholinergic activation underlying theta frequency downward regulation in novel environment is yet to be established. Theta oscillation correlates with a variety of behavior performances [71], likely through differential mechanisms that engage differential neuronal populations and neurotransmitters [9]. Understanding the mechanisms underlying such regulation will greatly improve our understanding of theta generation and how theta oscillations can contribute to various behavioral outcomes.

## 6. Cholinergic Regulation of Theta-Gamma Coupling

In addition to theta oscillations, there are gamma oscillations in the hippocampus with a frequency ranging from 25 to 100 Hz [72]. Gamma oscillations co-occur with theta oscillations. Both gamma amplitude and phase can be coupled to theta phase [73]. Within gamma oscillations, two frequency bands are further differentiated, namely slow gamma (25 to 60 Hz) and fast gamma (60 to 100 Hz) [7,72]. CA1 slow gamma is coherent with CA3 slow gamma and thus may facilitate information flow from CA3 to CA1 and memory retrieval, while fast gamma in CA1 is synchronized with fast gamma in the medial entorhinal cortex and thus may facilitate entorhinal inputs to CA1 and memory encoding [7,9,68,72,74,75,76,77,78]. Fast and slow gamma also occurred in different phases of theta rhythm, suggesting a mechanism for theta rhythm to segregate information flow to CA1 from the medial entorhinal cortex and CA3 and associated memory encoding and retrieval processes, respectively. Theta-gamma coupling has been associated with several types of learning processes [7,77,79,80,81], and reduced theta-gamma coupling is associated with cognitive impairments including those in aging and AD [82,83,84,85]. It is then not surprising that cholinergic transmission can also modulate theta-gamma coupling and subsequent behavior performance.

Recordings from the CA1 area of freely moving mice show that gamma activity is amplitude-modulated at θ frequencies. This theta-gamma coupling is stronger during active exploration than during awake immobility. Intraperitoneally administrated atropine increased theta irregularity, reduced gamma power and theta-gamma coupling during explorative behavior [86]. Stronger theta-gamma coupling during active exploration is also observed in the superficial layers of the medial entorhinal cortex of freely moving rats (Newman, 2013). Systemically administered scopolamine significantly reduced the coupling of theta and fast gamma (60 to 120 Hz) by primarily reducing fast gamma power at the peak of theta, shifting the peak gamma to occur at later theta phases [68]. There is also a slow gamma band (20 to 40 Hz) in the medial entorhinal cortex which is coupled to a theta phase that is about a quarter of theta cycle away from fast gamma. The slow gamma-theta coupling is also significantly upregulated by movement but is not significantly impacted by scopolamine [68]. This observation is consistent with the notion that both cholinergic transmission and fast gamma-theta coupling in CA1 and the medial entorhinal cortex are closely associated with memory encoding.

The mechanism underlying this cholinergic modulation of theta-gamma coupling is not clear. Studies suggest that hippocampal interneurons are critical for gamma generation. Slow gamma is driven by interneurons that are activated by CA3 inputs, while fast gamma is driven by interneurons activated by medial entorhinal cortical inputs [72,74,76,87]. Cholinergic inputs may thus target these interneurons to modulate gamma oscillations and theta-gamma coupling. Gamma oscillations and theta-gamma coupling also occur in frontal cortex. A recent study shows a clear contribution of cholinergic activation in prefrontal cortex to theta-gamma coupling during cue detection in rats [88]. Detected cues evoke transient increases in acetylcholine release in prefrontal cortex, which coincides with the emergence of theta-gamma coupling. The coupling occurs specifically between theta and slow gamma (47 to 57 Hz) but not between theta and fast gamma (75 to 90 Hz). Nicotinic receptors primarily contributed to high-gamma oscillations that occurred during the earliest phases of the cue detection process, while M1 muscarinic receptors contributed to the transition from high to low gamma power during cue-guided decision making [88]. Both M1 muscarinic and nicotinic receptors contributed to theta-gamma coupling, suggesting that the emergence of early high gamma is necessary for the later coupling of theta and low gamma. A computational modeling study further suggests that spatially constrained cholinergic activation can promote theta-gamma coupling in biophysically based excitatory-inhibitory neural network models through modulation of muscarinic receptor-regulated K^+^ current [89]. Gamma activity arises in regions with high ACh levels, while theta or mixed theta-gamma activity occurs at the peripheries of these high ACh regions. High gamma activity can also alternate between different high ACh regions at theta frequency [89], suggesting a causal role of cholinergic activity in promoting theta-gamma coupling.

## 7. Concluding Remarks and Future Perspectives

**Figure 1:**
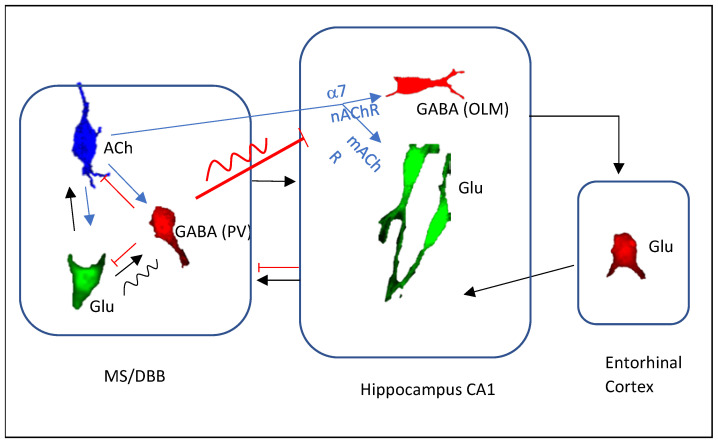
Cholinergic regulation of hippocampal theta oscillations. Hippocampal theta oscillations result from the interaction between the septohippocampal circuit that is critical for theta rhythm generation and the entorhinal-hippocampal circuit that is critical for theta amplitude generation. Cholinergic transmission primarily targets the septohippocampal circuit to regulate hippocampal theta oscillations directly through the septohippocampal cholinergic pathway or through septal local glutamatergic and GABAergic neurons. ACh, cholinergic transmission (blue lines); Glu, glutamatergic transmission (black lines); GABA, GABAergic transmission (red lines). Wavy lines indicate the ability to pace hippocampal theta oscillations.

Septal cholinergic neurons are activated during both type II and type I theta states. Cholinergic activation can regulate hippocampal theta through both direct septohippocampal cholinergic pathway and indirect local septal circuit (Figure 1). Elevated hippocampal ACh release during theta states can increase theta power through both mAChRs and nAChRs located at excitatory and inhibitory neurons, respectively. Septohippocampal cholinergic projection may also facilitate theta initiation by activating hippocampal theta-on cells, promote place cell phase precession and theta sequence stabilization through modulation of hippocampal synaptic transmission and plasticity, and hippocampal interaction with entorhinal inputs. Septal cholinergic activation can also act on local septal glutamatergic and GABAergic neurons to regulation theta frequency and power. Coordinated cholinergic action on hippocampal circuit and entorhinal inputs to the hippocampus may provide a mechanism to precisely regulate the timing of hippocampal pyramidal cell firing relative to theta phase which may play a crucial role in spatial memory encoding.

Local septal cholinergic action is essential for type II theta generation but is less important for type I theta and usually only has a moderate effect on type I theta frequency regulation. The relationship between type II theta centered on septal hippocampal loop and type I theta centered on the entorhinal hippocampal loop is currently unclear. Some researchers consider that type II theta coexists with type I theta in behaving animals and is only unmasked by anesthesia administration. It is also possible that each type of theta only occurs during its preferential states. Even if type II and type I theta coexist in behaving animals, it is still not clear if and how they are integrated to modify various aspects of behavior outcomes. Current studies seem to suggest that type II theta is more related to internal drive arising from past experiences, and type I theta is more related to external drive arising from ongoing activities.

Cholinergic transmission can target both glutamatergic and GABAergic neurons in MSDB and the hippocampus through both mAChRs and nAChRs. It is currently unclear if septal cholinergic activation directly regulates septal GABAergic neurons or indirectly through local glutamatergic neurons, or both, and what cholinergic receptor subtypes underlie such mechanisms, respectively. An Intersectional Recombinases-mediated Area-and-cell-Specific gene Excision (IRASE) method has recently been developed to achieve receptor knockout with spatial and cell-type specificity [90]. In this IRASE method, Flpo-expressing mouse lines are crossed with receptor floxed mouse lines and Cre is later introduced by injecting Flpo-dependent Cre-expressing viruses into a specific brain subregion. With the adoption of IRASE strategy, and the availability of a variety of Flpo expressing mouse lines [91], clarification of subregional contributions of cholinergic receptor subtypes and neuronal subpopulations to theta regulation can be expected in the near future.

## Figures and Tables

**Table 1:** Comparison of type I and type II theta oscillations.

	Type I Theta	Type II Theta
Occurrence	Active exploration	Urethane anesthesia; alert immobility
Theta frequency	6–12 Hz	4–9 Hz
Atropine dependence	Atropine-resistant	Atropine sensitive
MS-DBB dependence	Yes	Yes
EC dependence	Yes	No
NMDAR dependence	Yes	No

## References

[1] Witter M.P., Naber P.A., van Haeften T., Machielsen W.C., Rombouts S.A., Barkhof F., Scheltens P., Lopes da Silva F.H. (2000). Cortico-hippocampal communication by way of parallel parahippocampal-subicular pathways. Hippocampus.

[2] Amaral D.G., Scharfman H.E., Lavenex P. (2007). The dentate gyrus: Fundamental neuroanatomical organization (dentate gyrus for dummies). Prog. Brain Res..

[3] Zhang Y., Cao L., Varga V., Jing M., Karadas M., Li Y., Buzsaki G. (2021). Cholinergic suppression of hippocampal sharp-wave ripples impairs working memory. Proc. Natl. Acad. Sci. USA.

[4] Hasselmo M.E. (2006). The role of acetylcholine in learning and memory. Curr. Opin. Neurobiol..

[5] Zheng C., Hwaun E., Loza C.A., Colgin L.L. (2021). Hippocampal place cell sequences differ during correct and error trials in a spatial memory task. Nat. Commun..

[6] Qasim S.E., Fried I., Jacobs J. (2021). Phase precession in the human hippocampus and entorhinal cortex. Cell.

[7] Nunez A., Buno W. (2021). The Theta Rhythm of the Hippocampus: From Neuronal and Circuit Mechanisms to Behavior. Front. Cell Neurosci..

[8] Buzsaki G., Moser E.I. (2013). Memory, navigation and theta rhythm in the hippocampal-entorhinal system. Nat. Neurosci..

[9] Lopez-Madrona V.J., Perez-Montoyo E., Alvarez-Salvado E., Moratal D., Herreras O., Pereda E., Mirasso C.R., Canals S. (2020). Different theta frameworks coexist in the rat hippocampus and are coordinated during memory-guided and novelty tasks. eLife.

[10] Hasselmo M.E. (2005). What is the function of hippocampal theta rhythm?—Linking behavioral data to phasic properties of field potential and unit recording data. Hippocampus.

[11] Kramis R., Vanderwolf C.H., Bland B.H. (1975). Two types of hippocampal rhythmical slow activity in both the rabbit and the rat: Relations to behavior and effects of atropine, diethyl ether, urethane, and pentobarbital. Exp. Neurol..

[12] Buzsaki G. (2002). Theta oscillations in the hippocampus. Neuron.

[13] Gu Z., Alexander G.M., Dudek S.M., Yakel J.L. (2017). Hippocampus and Entorhinal Cortex Recruit Cholinergic and NMDA Receptors Separately to Generate Hippocampal Theta Oscillations. Cell Rep..

[14] Gu Z., Smith K.G., Alexander G.M., Guerreiro I., Dudek S.M., Gutkin B., Jensen P., Yakel J.L. (2020). Hippocampal Interneuronal alpha7 nAChRs Modulate Theta Oscillations in Freely Moving Mice. Cell Rep..

[15] Dannenberg H., Pabst M., Braganza O., Schoch S., Niediek J., Bayraktar M., Mormann F., Beck H. (2015). Synergy of direct and indirect cholinergic septo-hippocampal pathways coordinates firing in hippocampal networks. J. Neurosci..

[16] Vandecasteele M., Varga V., Berenyi A., Papp E., Bartho P., Venance L., Freund T.F., Buzsaki G. (2014). Optogenetic activation of septal cholinergic neurons suppresses sharp wave ripples and enhances theta oscillations in the hippocampus. Proc. Natl. Acad. Sci. USA.

[17] Quirk C.R., Zutshi I., Srikanth S., Fu M.L., Devico Marciano N., Wright M.K., Parsey D.F., Liu S., Siretskiy R.E., Huynh T.L. (2021). Precisely timed theta oscillations are selectively required during the encoding phase of memory. Nat. Neurosci..

[18] King C., Recce M., O’Keefe J. (1998). The rhythmicity of cells of the medial septum/diagonal band of Broca in the awake freely moving rat: Relationships with behaviour and hippocampal theta. Eur. J. Neurosci..

[19] Zhang H., Lin S.C., Nicolelis M.A. (2010). Spatiotemporal coupling between hippocampal acetylcholine release and theta oscillations in vivo. J. Neurosci..

[20] Marrosu F., Portas C., Mascia M.S., Casu M.A., Fa M., Giagheddu M., Imperato A., Gessa G.L. (1995). Microdialysis measurement of cortical and hippocampal acetylcholine release during sleep-wake cycle in freely moving cats. Brain Res..

[21] Ma X., Zhang Y., Wang L., Li N., Barkai E., Zhang X., Lin L., Xu J. (2020). The Firing of Theta State-Related Septal Cholinergic Neurons Disrupt Hippocampal Ripple Oscillations via Muscarinic Receptors. J. Neurosci..

[22] Giovannini M.G., Rakovska A., Benton R.S., Pazzagli M., Bianchi L., Pepeu G. (2001). Effects of novelty and habituation on acetylcholine, GABA, and glutamate release from the frontal cortex and hippocampus of freely moving rats. Neuroscience.

[23] Bianchi L., Ballini C., Colivicchi M.A., Della Corte L., Giovannini M.G., Pepeu G. (2003). Investigation on acetylcholine, aspartate, glutamate and GABA extracellular levels from ventral hippocampus during repeated exploratory activity in the rat. Neurochem. Res..

[24] Gu Z., Yakel J.L. (2017). Inducing theta oscillations in the entorhinal hippocampal network in vitro. Brain Struct. Funct..

[25] Stoiljkovic M., Kelley C., Nagy D., Leventhal L., Hajos M. (2016). Selective activation of alpha7 nicotinic acetylcholine receptors augments hippocampal oscillations. Neuropharmacology.

[26] Siok C.J., Rogers J.A., Kocsis B., Hajos M. (2006). Activation of alpha7 acetylcholine receptors augments stimulation-induced hippocampal theta oscillation. Eur. J. Neurosci..

[27] Cobb S.R., Bulters D.O., Suchak S., Riedel G., Morris R.G., Davies C.H. (1999). Activation of nicotinic acetylcholine receptors patterns network activity in the rodent hippocampus. J. Physiol..

[28] Letsinger A.C., Gu Z., Yakel J.L. (2022). alpha7 nicotinic acetylcholine receptors in the hippocampal circuit: Taming complexity. Trends Neurosci..

[29] Haam J., Zhou J., Cui G., Yakel J.L. (2018). Septal cholinergic neurons gate hippocampal output to entorhinal cortex via oriens lacunosum moleculare interneurons. Proc. Natl. Acad. Sci. USA.

[30] Siwani S., Franca A.S.C., Mikulovic S., Reis A., Hilscher M.M., Edwards S.J., Leao R.N., Tort A.B.L., Kullander K. (2018). OLMalpha2 Cells Bidirectionally Modulate Learning. Neuron.

[31] Sekulic V., Yi F., Garrett T., Guet-McCreight A., Lawrence J.J., Skinner F.K. (2020). Integration of Within-Cell Experimental Data With Multi-Compartmental Modeling Predicts H-Channel Densities and Distributions in Hippocampal OLM Cells. Front. Cell Neurosci..

[32] Salimi-Nezhad N., Hasanlou M., Amiri M., Keliris G.A. (2021). A neuromimetic realization of hippocampal CA1 for theta wave generation. Neural Netw..

[33] Leao R.N., Mikulovic S., Leao K.E., Munguba H., Gezelius H., Enjin A., Patra K., Eriksson A., Loew L.M., Tort A.B. (2012). OLM interneurons differentially modulate CA3 and entorhinal inputs to hippocampal CA1 neurons. Nat. Neurosci..

[34] Mikulovic S., Restrepo C.E., Siwani S., Bauer P., Pupe S., Tort A.B.L., Kullander K., Leao R.N. (2018). Ventral hippocampal OLM cells control type 2 theta oscillations and response to predator odor. Nat. Commun..

[35] Zhou H., Neville K.R., Goldstein N., Kabu S., Kausar N., Ye R., Nguyen T.T., Gelwan N., Hyman B.T., Gomperts S.N. (2019). Cholinergic modulation of hippocampal calcium activity across the sleep-wake cycle. eLife.

[36] Cohen J.D., Bolstad M., Lee A.K. (2017). Experience-dependent shaping of hippocampal CA1 intracellular activity in novel and familiar environments. eLife.

[37] Dragoi G., Buzsaki G. (2006). Temporal encoding of place sequences by hippocampal cell assemblies. Neuron.

[38] Pastalkova E., Itskov V., Amarasingham A., Buzsaki G. (2008). Internally generated cell assembly sequences in the rat hippocampus. Science.

[39] Venditto S.J.C., Le B., Newman E.L. (2019). Place cell assemblies remain intact, despite reduced phase precession, after cholinergic disruption. Hippocampus.

[40] Newman E.L., Venditto S.J.C., Climer J.R., Petter E.A., Gillet S.N., Levy S. (2017). Precise spike timing dynamics of hippocampal place cell activity sensitive to cholinergic disruption. Hippocampus.

[41] Schlesiger M.I., Cannova C.C., Boublil B.L., Hales J.B., Mankin E.A., Brandon M.P., Leutgeb J.K., Leibold C., Leutgeb S. (2015). The medial entorhinal cortex is necessary for temporal organization of hippocampal neuronal activity. Nat. Neurosci..

[42] Feng T., Silva D., Foster D.J. (2015). Dissociation between the experience-dependent development of hippocampal theta sequences and single-trial phase precession. J. Neurosci..

[43] Dragoi G., Tonegawa S. (2013). Distinct preplay of multiple novel spatial experiences in the rat. Proc. Natl. Acad. Sci. USA.

[44] Dragoi G., Tonegawa S. (2011). Preplay of future place cell sequences by hippocampal cellular assemblies. Nature.

[45] Rogers J.L., Kesner R.P. (2003). Cholinergic modulation of the hippocampus during encoding and retrieval. Neurobiol. Learn. Mem..

[46] Yoder R.M., Pang K.C. (2005). Involvement of GABAergic and cholinergic medial septal neurons in hippocampal theta rhythm. Hippocampus.

[47] Bland B.H., Oddie S.D., Colom L.V. (1999). Mechanisms of neural synchrony in the septohippocampal pathways underlying hippocampal theta generation. J. Neurosci..

[48] Lee M.G., Chrobak J.J., Sik A., Wiley R.G., Buzsaki G. (1994). Hippocampal theta activity following selective lesion of the septal cholinergic system. Neuroscience.

[49] Simon A.P., Poindessous-Jazat F., Dutar P., Epelbaum J., Bassant M.H. (2006). Firing properties of anatomically identified neurons in the medial septum of anesthetized and unanesthetized restrained rats. J. Neurosci..

[50] Sotty F., Danik M., Manseau F., Laplante F., Quirion R., Williams S. (2003). Distinct electrophysiological properties of glutamatergic, cholinergic and GABAergic rat septohippocampal neurons: Novel implications for hippocampal rhythmicity. J. Physiol..

[51] Lawson V.H., Bland B.H. (1993). The role of the septohippocampal pathway in the regulation of hippocampal field activity and behavior: Analysis by the intraseptal microinfusion of carbachol, atropine, and procaine. Exp. Neurol..

[52] Broncel A., Bocian R., Klos-Wojtczak P., Konopacki J. (2018). Medial septal cholinergic mediation of hippocampal theta rhythm induced by vagal nerve stimulation. PLoS ONE.

[53] Oddie S.D., Bland B.H. (1998). Hippocampal formation theta activity and movement selection. Neurosci. Biobehav. Rev..

[54] Golebiewski H., Eckersdorf B., Konopacki J. (2002). Septal cholinergic mediation of hippocampal theta in the cat. Brain Res. Bull..

[55] Li S., Topchiy I., Kocsis B. (2007). The effect of atropine administered in the medial septum or hippocampus on high- and low-frequency theta rhythms in the hippocampus of urethane anesthetized rats. Synapse.

[56] Smythe J.W., Colom L.V., Bland B.H. (1992). The extrinsic modulation of hippocampal theta depends on the coactivation of cholinergic and GABA-ergic medial septal inputs. Neurosci. Biobehav. Rev..

[57] Manseau F., Goutagny R., Danik M., Williams S. (2008). The hippocamposeptal pathway generates rhythmic firing of GABAergic neurons in the medial septum and diagonal bands: An investigation using a complete septohippocampal preparation in vitro. J. Neurosci..

[58] Robinson J., Manseau F., Ducharme G., Amilhon B., Vigneault E., El Mestikawy S., Williams S. (2016). Optogenetic Activation of Septal Glutamatergic Neurons Drive Hippocampal Theta Rhythms. J. Neurosci..

[59] Ibrahim K.M., Ariffin M.Z., Khanna S. (2021). Modulation of Septo-Hippocampal Neural Responses in Anesthetized and Behaving Rats by Septal AMPA Receptor Mechanisms. Front. Neural Circuits.

[60] Manseau F., Danik M., Williams S. (2005). A functional glutamatergic neurone network in the medial septum and diagonal band area. J. Physiol..

[61] Puma C., Bizot J.C. (1999). Hippocampal theta rhythm in anesthetized rats: Role of AMPA glutamate receptors. Neuroreport.

[62] Fuhrmann F., Justus D., Sosulina L., Kaneko H., Beutel T., Friedrichs D., Schoch S., Schwarz M.K., Fuhrmann M., Remy S. (2015). Locomotion, Theta Oscillations, and the Speed-Correlated Firing of Hippocampal Neurons Are Controlled by a Medial Septal Glutamatergic Circuit. Neuron.

[63] Jeewajee A., Lever C., Burton S., O’Keefe J., Burgess N. (2008). Environmental novelty is signaled by reduction of the hippocampal theta frequency. Hippocampus.

[64] Wells C.E., Amos D.P., Jeewajee A., Douchamps V., Rodgers J., O’Keefe J., Burgess N., Lever C. (2013). Novelty and anxiolytic drugs dissociate two components of hippocampal theta in behaving rats. J. Neurosci..

[65] Jeewajee A., Barry C., O’Keefe J., Burgess N. (2008). Grid cells and theta as oscillatory interference: Electrophysiological data from freely moving rats. Hippocampus.

[66] Bush D., Bisby J.A., Bird C.M., Gollwitzer S., Rodionov R., Diehl B., McEvoy A.W., Walker M.C., Burgess N. (2017). Human hippocampal theta power indicates movement onset and distance travelled. Proc. Natl. Acad. Sci. USA.

[67] Kropff E., Carmichael J.E., Moser E.I., Moser M.B. (2021). Frequency of theta rhythm is controlled by acceleration, but not speed, in running rats. Neuron.

[68] Newman E.L., Gillet S.N., Climer J.R., Hasselmo M.E. (2013). Cholinergic blockade reduces theta-gamma phase amplitude coupling and speed modulation of theta frequency consistent with behavioral effects on encoding. J. Neurosci..

[69] Givens B., Olton D.S. (1995). Bidirectional modulation of scopolamine-induced working memory impairments by muscarinic activation of the medial septal area. Neurobiol. Learn. Mem..

[70] Thiel C.M., Huston J.P., Schwarting R.K. (1998). Hippocampal acetylcholine and habituation learning. Neuroscience.

[71] Korotkova T., Ponomarenko A., Monaghan C.K., Poulter S.L., Cacucci F., Wills T., Hasselmo M.E., Lever C. (2018). Reconciling the different faces of hippocampal theta: The role of theta oscillations in cognitive, emotional and innate behaviors. Neurosci. Biobehav. Rev..

[72] Colgin L.L. (2016). Rhythms of the hippocampal network. Nat. Rev. Neurosci..

[73] Belluscio M.A., Mizuseki K., Schmidt R., Kempter R., Buzsaki G. (2012). Cross-frequency phase-phase coupling between theta and gamma oscillations in the hippocampus. J. Neurosci..

[74] Schomburg E.W., Fernandez-Ruiz A., Mizuseki K., Berenyi A., Anastassiou C.A., Koch C., Buzsaki G. (2014). Theta phase segregation of input-specific gamma patterns in entorhinal-hippocampal networks. Neuron.

[75] Hasselmo M.E., Bodelon C., Wyble B.P. (2002). A proposed function for hippocampal theta rhythm: Separate phases of encoding and retrieval enhance reversal of prior learning. Neural Comput..

[76] Colgin L.L., Denninger T., Fyhn M., Hafting T., Bonnevie T., Jensen O., Moser M.B., Moser E.I. (2009). Frequency of gamma oscillations routes flow of information in the hippocampus. Nature.

[77] Lisman J.E., Jensen O. (2013). The theta-gamma neural code. Neuron.

[78] Colgin L.L. (2015). Theta-gamma coupling in the entorhinal-hippocampal system. Curr. Opin. Neurobiol..

[79] Tort A.B., Komorowski R.W., Manns J.R., Kopell N.J., Eichenbaum H. (2009). Theta-gamma coupling increases during the learning of item-context associations. Proc. Natl. Acad. Sci. USA.

[80] Tort A.B., Kramer M.A., Thorn C., Gibson D.J., Kubota Y., Graybiel A.M., Kopell N.J. (2008). Dynamic cross-frequency couplings of local field potential oscillations in rat striatum and hippocampus during performance of a T-maze task. Proc. Natl. Acad. Sci. USA.

[81] Axmacher N., Henseler M.M., Jensen O., Weinreich I., Elger C.E., Fell J. (2010). Cross-frequency coupling supports multi-item working memory in the human hippocampus. Proc. Natl. Acad. Sci. USA.

[82] Goodman M.S., Kumar S., Zomorrodi R., Ghazala Z., Cheam A.S.M., Barr M.S., Daskalakis Z.J., Blumberger D.M., Fischer C., Flint A. (2018). Theta-Gamma Coupling and Working Memory in Alzheimer’s Dementia and Mild Cognitive Impairment. Front. Aging Neurosci..

[83] Musaeus C.S., Nielsen M.S., Musaeus J.S., Hogh P. (2020). Electroencephalographic Cross-Frequency Coupling as a Sign of Disease Progression in Patients With Mild Cognitive Impairment: A Pilot Study. Front. Neurosci..

[84] Jacobson T.K., Howe M.D., Schmidt B., Hinman J.R., Escabi M.A., Markus E.J. (2013). Hippocampal theta, gamma, and theta-gamma coupling: Effects of aging, environmental change, and cholinergic activation. J. Neurophysiol..

[85] Goutagny R., Gu N., Cavanagh C., Jackson J., Chabot J.G., Quirion R., Krantic S., Williams S. (2013). Alterations in hippocampal network oscillations and theta-gamma coupling arise before Abeta overproduction in a mouse model of Alzheimer’s disease. Eur. J. Neurosci..

[86] Hentschke H., Perkins M.G., Pearce R.A., Banks M.I. (2007). Muscarinic blockade weakens interaction of gamma with theta rhythms in mouse hippocampus. Eur. J. Neurosci..

[87] Lasztoczi B., Klausberger T. (2014). Layer-specific GABAergic control of distinct gamma oscillations in the CA1 hippocampus. Neuron.

[88] Howe W.M., Gritton H.J., Lusk N.A., Roberts E.A., Hetrick V.L., Berke J.D., Sarter M. (2017). Acetylcholine Release in Prefrontal Cortex Promotes Gamma Oscillations and Theta-Gamma Coupling during Cue Detection. J. Neurosci..

[89] Yang Y., Gritton H., Sarter M., Aton S.J., Booth V., Zochowski M. (2021). Theta-gamma coupling emerges from spatially heterogeneous cholinergic neuromodulation. PLoS Comput. Biol..

[90] Penzo M.A., Robert V., Tucciarone J., De Bundel D., Wang M., Van Aelst L., Darvas M., Parada L.F., Palmiter R.D., He M. (2015). The paraventricular thalamus controls a central amygdala fear circuit. Nature.

[91] Daigle T.L., Madisen L., Hage T.A., Valley M.T., Knoblich U., Larsen R.S., Takeno M.M., Huang L., Gu H., Larsen R. (2018). A Suite of Transgenic Driver and Reporter Mouse Lines with Enhanced Brain-Cell-Type Targeting and Functionality. Cell.

